# Genomics control of biostimulant‐induced stress tolerance and crop yield enhancement

**DOI:** 10.1111/tpj.70382

**Published:** 2025-07-26

**Authors:** Tsanko Gechev

**Affiliations:** ^1^ Department of Molecular Stress Physiology Center of Plant Systems Biology and Biotechnology 14 Knyaz Boris I Pokrastitel Plovdiv 4023 Bulgaria; ^2^ Department of Molecular Biology University of Plovdiv Plovdiv 4000 Bulgaria

**Keywords:** abiotic stress, Arabidopsis, biostimulants, drought, oxidative stress, tomato

## Abstract

Biostimulants are changing modern agriculture, as they have the potential to secure healthy and sustainable food production while preserving the environment. They have two main biological effects: growth promotion and stress protection. Both effects can lead to enhancement of the yield and improvement of the marketable grade of the produce in crops, without compromising crop quality. Their use increased exponentially in the past decade, as they are highly efficient, ecologically friendly (non‐toxic, biodegradable), and applicable to all major crops. While exponential data on the physiological mechanisms of stress protection is accumulating in recent years, the information as to how biostimulants act at the molecular level is still rather limited. Here we review the growing evidence of the biostimulants role in stress protection and yield enhancement of crops, as well as the recent transcriptomic and metabolomic data, which indicate biostimulants' molecular mode of action. In particular, we outline the role of genes encoding signaling components, plant hormones (abscisic acid, brassinosteroids, and ethylene), genes encoding transcription factors from ERF, WRKY, NAC, and MYB families, and genes related to growth, photosynthesis, and stress response. Finally, we describe strategies to study the genetic and genomics control of biostimulants mode of action, with foci on stress tolerance and yield enhancement. In Arabidopsis, established systems for biostimulants‐induced protection against drought and oxidative stress will allow both forward and reverse genetics approaches to identify key genes from the biostimulants network. Mutations in such genes compromise the stress‐protective effect of biostimulants. In major crops such as pepper and tomato, large Genome Wide Association Studies (GWAS) panels can be utilized to study crops responses to biostimulants in terms of drought tolerance, fruit qualities, and yield in order to pinpoint genes controlling biostimulants‐induced stress protection and yield enhancement. The combination of these approaches allows identification and verification of important genes involved in the pathways of biostimulant‐induced stress protection and yield enhancement, as well as deciphering parts of the intricate biostimulant‐signaling network.

## Significance Statement

The article reviews the latest research on plant biostimulants, with a focus on physiological and molecular mechanisms, which regulate development and stress responses. It also describes strategies to identify new genes of the stress network modulated by biostimulants.

## INTRODUCTION

Abiotic stresses such as drought, salinity, extreme temperatures, and pollutants cause significant reductions in growth, yield, and marketable grade of crops, which can account for up to 80% of global crop yield losses (Sujeeth et al., 2022). These adverse environmental conditions not only damage plants directly, but they also increase crop susceptibility to pests and diseases (Velásquez et al., 2018).

Biostimulants are naturally occurring molecules or microorganisms with growth promotion and stress protection activities. The most common groups of biostimulants on the market are derived from seaweeds (33% of the global market), followed by humic acids, fulvic acids, protein hydrolysates, amino acids, and microorganisms (Sujeeth et al., 2022).

Seaweed biostimulants can activate the defense mechanisms of plants, a process referred to as molecular priming, and protect from adverse environmental conditions (Kerchev et al., 2020). For example, biostimulants produced by the brown algae *Ascophyllum nodosum* can protect *Arabidopsis thaliana*, pepper, rice, and tomato from oxidative stress; Arabidopsis, *Brassica juncea*, maize, okra, olive, strawberry, sugarcane, and tomato from drought; papaya and raspberry from heat; tomato from cold stress; and rice from heavy metals and salt stress (Ali et al., 2022; Borella et al., 2023; Dias et al., 2024; Ferreira et al., 2025; Hasanuzzaman, Raihan, Nowroz, & Nahar, 2023; Hasanuzzaman, Raihan, Siddika, et al., 2023; Jacomassi et al., 2022; Kanojia et al., 2024; Omidbakhshfard et al., 2020; Pereira et al., 2025; Rasul et al., 2021; Shahzad et al., 2023; Staykov et al., 2020; Sujata et al., 2023) (Table 1). Moreover, seaweed biostimulants can stimulate growth, enhance yield, and improve the marketable grade of fruits and vegetables without compromising crop quality, therefore contributing to a sustainable agriculture. This has recently been documented for berry, cereal, and vegetable crops such as grapevine, raspberry, strawberry, sweet cherry, and tomato (Borella et al., 2023; Goñi et al., 2021; Kanojia et al., 2024; Kazakov et al., 2024; Łangowski et al., 2022; Monteiro et al., 2023; Monteiro et al., 2024; Pereira, Rodrigues, et al., 2024; Pereira, Silva, et al., 2024) (Table 1). The advantages of the seaweed biostimulants‐induced molecular priming technology can be summarized as follows: (i) Seaweed biostimulants are highly efficient: small amounts of seaweed‐derived biostimulants can trigger protection from abiotic and oxidative stresses such as drought, heat, and salinity and significant yield enhancement (42% in raspberry, 33% in strawberry, 70% in tomato, compared with untreated plants) (Table 1) (Dias et al., 2024; Ferreira et al., 2025; Kanojia et al., 2024; Kazakov et al., 2024; Omidbakhshfard et al., 2020; Rasul et al., 2021; Shahzad et al., 2023; Staykov et al., 2020); (ii) Biostimulants can improve marketable grade and nutritional properties: larger, more uniform berry and vegetable fruits; raspberries and strawberries richer in essential nutrients such as magnesium (Kanojia et al., 2024; Kazakov et al., 2024); (iii) The molecular priming technology based on biostimulants is ecologically friendly and sustainable. As seaweed biostimulants derive from natural products (e.g., brown algae such as *Ascophyllum nodosum*), they are non‐toxic, biodegradable, and do not pollute the environment. Furthermore, they reduce the use of traditional agrochemicals. They are obtained by controlled harvesting, preserving the natural habitats and resources (Sujeeth et al., 2022); (iv) Biostimulants are easily applicable to all crops (berries, cereals, vegetable crops) by adding them into the soil, by foliar spray, or by seed priming.

**Table 1:** Biostimulants mitigate stresses and enhance crop yield

Crop	Biological effect on the crop resulting from the biostimulant	Biostimulant	References
Apricot	Enhanced secondary metabolism, higher polyphenol, flavonoid, and anthocyanins.	Seaweed and yeast extracts	Gatti et al. (2025)
Barley	Improved nitrogen use efficiency.	*A. nodosum*‐derived biostimulant	Goñi et al. (2021)
*Brassica juncea*	Better performance under drought.	*A. nodosum*‐derived biostimulant	Sujata et al. (2023)
Cotton	Tolerance to drought stress. Detoxification of cadmium, higher yield.	γ‐polyglutamic acid; Combined biochar, microbial, and algal biostimulants	Wang et al. (2025), Osman et al. (2025)
Cucumber	Growth promotion and defense against pathogens.	Oligosaccharides	Pring et al. (2025)
Grapevine	Enhancement of berry quality: Increase of antioxidant activity, as well as increase in anthocyanins and other phenolics.	*A. nodosum* extract, glycine betaine	Monteiro et al. (2024), Monteiro et al. (2023)
Kiwifruit	Improved growth and rooting.	*A. nodosum*‐derived biostimulant	Dutta et al. (2023)
Lavender	Enhancing growth and essential oil components.	Humic acid and extract from *Malva parviflora*	El‐Hefny and Hussien (2025)
Lettuce	Increased growth and biomass under salt stress.	*A. nodosum*‐derived biostimulant	Guinan et al. (2013)
Maize	Enhanced biomass and drought tolerance. Increased biomass, photosynthetic pigments, and antioxidant enzyme activities under cadmium stress.	Chitosan, fulvic acid, and their combination; Humic acid	Brown et al. (2024), Mishra et al. (2025), Song et al. (2025)
Okra	Improved biochemical and physiological functions under drought.	*A. nodosum*‐derived biostimulant	Ali et al. (2022)
Olive	Improved growth and photosynthesis under drought.	*A. nodosum*‐derived biostimulant	Dias et al. (2024)
Papaya	Better growth under heat.	*A. nodosum*‐derived biostimulant	Ferreira et al. (2025)
Pepper	Tolerance to oxidative stress.	*A. nodosum*‐derived biostimulant	Staykov et al. (2021)
Purslane	Tolerance to salt stress.	Putrescine	Mohamed et al. (2024)
Raspberry	42.1% increase in yield of the primed plants, compared with the unprimed, and higher Mg content. Mitigation of heat stress.	*A. nodosum*‐derived biostimulant	Kazakov et al. (2024), Makonya et al. (2025)
Rice	Enhanced salt tolerance. Mitigation of heavy metal (arsenic, cadmium) and oxidative stress.	*A. nodosum*‐derived biostimulant	Hasanuzzaman, Raihan, Siddika, et al. (2023), Hasanuzzaman, Raihan, Nowroz, and Nahar (2023), Shahzad et al. (2023)
Soybean	Enhanced growth and increased salt tolerance.	Chitosan‐fulvic acid	Huy et al. (2025)
Strawberry	Enhances salinity tolerance; Increases the yield and Mg content in fruits; Tolerance to drought.	Putrescine; extracts from *A. nodosum*	Muradoğlu et al. (2025), Kazakov et al. (2024), Alam et al. (2013), Pereira et al. (2025)
Sugarcane	Improved performance under drought.	*A. nodosum*‐derived biostimulant	Jacomassi et al. (2022)
Sweet cherry	Higher sugar and protein content, enhanced antioxidant activity and phenolic content.	Calcium and seaweed extracts	Pereira, Rodrigues, et al. (2024); Pereira, Silva, et al. (2024)
Tomato	Mitigation of osmotic and cold stress; Increased fruit number, enhanced drought tolerance; Enhanced salt stress tolerance and 70% yield enhancement.	Humic acid, protein hydrolysates, seaweed extracts	Borella et al. (2023), Lengrand et al. (2024), Kanojia et al. (2024), Di Stasio et al. (2020), Zhang et al. (2023)
Wheat	Tolerance to salinity. Improved photosynthesis, nitrogen use efficiency, and Na^+^/K^+^ ratio.	Humic acid, *A. nodosum* extracts	Alghabari and Shah (2025), Łangowski et al. (2022)

The other groups of biostimulants, such as humic acid, fulvic acid, glycine betaine, polyamines such as putrescine, spermidine, and spermine, and microbial biostimulants, can exert similar beneficial effects on plant growth and stress tolerance. Some of them are used either alone or in combination with *A. nodosum* extracts or other biostimulants. Humic acid, for example, protected wheat from salt stress by enhancing chlorophyll and photosynthesis, increasing the activities of antioxidant enzymes superoxide dismutase (SOD), peroxidase (POD), and catalase (CAT), upregulating salt stress‐related genes such as *TaNHX1, TaHKT1,4, TaAKT1, TaPRX2A*, *TaSOD*, and *TaCAT1*, and reducing the Na^+^/K^+^ ratio (Alghabari & Shah, 2025). Humic acid enhanced photosynthetic pigments, antioxidant enzyme activities, and biomass in maize grown on Cd contaminated soils (Song et al., 2025). In tomato, humic acid protects from osmotic stress (Lengrand et al., 2024). Furthermore, the combination of humic acid together with extracts from *Malva parviflora* enhances the growth and essential oils of *Lavandula latifolia* (El‐Hefny & Hussien, 2025). Combinations of two or more biostimulants proved synergistic on other occasions as well.

Chitosan nanoparticles releasing fulvic acid enhanced growth and mitigated salt stress in soybean (Huy et al., 2025). This was concomitant with higher chlorophyll under both control and salt conditions, activation of antioxidant enzymes, lower stress parameters such as malondialdehyde and H_2_O_2_ contents, and increased expression of the Na^+^ detoxifying genes *GmSOS1, GmSOS2, GmNHX1*, and *GmP5CS1* (Huy et al., 2025). Chitosan and fulvic acid alone, as well as chitosan‐fulvic acid nanoparticles, were successfully employed to enhance drought tolerance in maize, and the largest effect was observed when chitosan and fulvic acid were together (Brown et al., 2024).

Glycine betaine and extracts from *A. nodosum* were used to improve the quality of grapevine (Monteiro et al., 2023; Monteiro et al., 2024). Foliar preharvest application of 0.2% glycine betaine resulted in berries with higher anthocyanin and other phenolic levels, as well as with increased antioxidant activities (Monteiro et al., 2023; Monteiro et al., 2024). This was concomitant with the induction of genes involved in the synthesis and transport of anthocyanins (*CHALCONE SYNTHASE (CHS), FLAVANONE 3‐HYDROXYLASE (F3H), FLAVONOID GLYCOSYLTRANSFERASES (UFGT)*, and *GLUTATHIONE‐S‐TRANSFERASE (GST)*) (Monteiro et al., 2024).

Putrescine, a small polyamine, has important roles in plant development and stress responses. Recently, it was successfully used to enhance salt stress tolerance in Arabidopsis and strawberry (Jasso‐Robles et al., 2024; Muradoğlu et al., 2025). Putrescine is synthesized by the activity of arginine decarboxylase (ADC; EC: 4.1.1.17). It can be acetylated by enzymes such as SPERMINE/SPERMIDINE N‐ACETYLTRANSFERASES (SSATs) or N‐ACETYLTRANSFERASE ACTIVITIES (NATAs), which may be necessary for catabolism or membrane transport. Interestingly, loss of function of the Arabidopsis *AtNATA2* enhances putrescine biosynthesis through *AtADC2* and triggers putrescine molecular priming, which protects Arabidopsis from salt stress (Jasso‐Robles et al., 2024).

Microbial biostimulants, including plant growth‐promoting rhizobacteria, arbuscular mycorrhizal fungi, nitrogen‐fixing bacteria, and endophytes, gain more popularity as well (Ali et al., 2024; Pereira et al., 2025). They are totally different from the other types of biostimulants as they contain live microorganisms. Moreover, such biostimulants can contain even mixtures of different microorganisms. For example, a biostimulant derived from a mixture of mycorrhizal microorganisms and rhizobacteria exhibited a positive effect on the growth of tomato seedlings under standard conditions and under salt stress (Justamante et al., 2025). There are also examples of combined application of both plant and microbial biostimulants. For example, a biostimulant derived from the medicinal plant *Adathoda vasica*, together with plant growth‐promoting rhizobacteria, mitigated drought stress in maize (Mishra et al., 2025). Whereas there is no doubt that the microbial biostimulants can be beneficial for plant growth and stress tolerance, their safety should be rigorously assessed as they are intentionally released into the environment (Bellotti et al., 2025).

Biostimulants have different degrees of compositional complexity. Whereas some biostimulants have simple chemical compositions, such as chitosan, fulvic acid, or glycine betaine, other biostimulants, like the protein hydrolysates, are more complex, and the plant extracts and the microorganisms are the most complex of all. The composition of plant‐based biostimulants depends largely on the plant species but also on the technology of extraction, which is often proprietary to the producing company. The most commonly used seaweed biostimulants contain polysaccharides such as fucoidan, laminarin, alginates, as well as various phenolics, minerals, and other bioactive compounds (Sujeeth et al., 2022). In such complex mixtures, it is difficult to determine the most bioactive compound, and often there is a synergistic effect of several bioactive components from the complex mixture. For example, an oligosaccharide mixture (Oligo‐Mix) derived from plant cell walls and fungal cell walls stimulated growth in cucumber and provided protection against fungal pathogens (Pring et al., 2025). Microbial biostimulants, being the most complex of all, may act both through such oligosaccharide signals and through creating a beneficial growth‐promoting rhizosphere.

These positive effects of the biostimulant treatment are good for both the farmers and the end users, who will benefit from lower consumer prices and health‐stimulating properties of the fruits from primed crops (Di Gaudio et al., 2025; Kazakov et al., 2024). Due to the above‐mentioned advantages, the use of seaweed biostimulants is growing exponentially and its market exceeds 2.6 Bln EUR, with projections to further increase in the future (Sujeeth et al., 2022). The perspectives of using biostimulant‐based molecular priming are enormous and attract attention both from fundamental and applied research points of view (Di Sario et al., 2025). Despite the surge in using this technology and its success, there are limited studies on transcriptome and metabolome reprogramming during seaweed‐based molecular priming, and there are no functional studies, which confirm the participation of any of the regulated genes as part of the presumably complex biostimulant signaling network.

To address this gap, here we describe two alternative strategies (forward/reverse genetic screening and GWAS analysis, both described below in Section 3) to discover and functionally validate genes from the biostimulant‐controlled signaling network that control stress tolerance and yield.

## BIOSTIMULANT‐INDUCED MOLECULAR PRIMING TRIGGERS GLOBAL TRANSCRIPTOME REPROGRAMMING

Whereas the large majority of studies demonstrate mainly the physiological aspects of biostimulants‐induced growth and stress protection, a growing number of publications in recent years focuses also on the transcriptome and metabolome reconfigurations during the biostimulants‐induced molecular priming.

In Arabidopsis, comprehensive transcriptional profiling of *A. nodosum*‐derived molecular priming was performed during drought and oxidative stress (Omidbakhshfard et al., 2020; Rasul et al., 2021). The biostimulant‐induced molecular priming resulted in repression of the stress‐responsive negative growth regulator *RESPONSIVE TO DESICCATION 26* (*RD26*) at the shoot apical meristem. At the same time, the expression of the cell cycle marker gene *HISTONE H4* (*HIS4*), which is normally repressed by drought, was maintained in the shoot apical meristem in the biostimulant‐primed plants under drought conditions (Rasul et al., 2021). Accordingly, the expression of two other cell cycle genes, *CYCP2;1* and *CYCA3;2*, repressed by drought stress, was upregulated by the biostimulant. *CYCP2;1* is a cyclin gene involved in the integration of genetic and nutritional information to promote meristem cell division in Arabidopsis, and the *CYCA3;2* gene encodes a cyclin‐dependent protein kinase that controls cell proliferation in meristems (Harashima et al., 2013; Takahashi et al., 2010). All this is consistent with the model that the biostimulant‐induced molecular priming maintains the activity of the shoot apical meristem during drought stress, enabling growth even under drought conditions. Photosynthesis‐related genes were either maintained or upregulated by the biostimulant treatment under both normal and drought conditions, indicating that strengthening photosynthesis is an integral part of the biostimulants mode of action (Omidbakhshfard et al., 2020; Rasul et al., 2021). Other genes commonly upregulated by the biostimulant in the two different experiments include signaling components (*CYSTEINE‐RICH RECEPTOR‐LIKE PROTEIN KINASE 31, CYSTEINE‐RICH RECEPTOR‐LIKE PROTEIN KINASE 37*, *Ca* ^*2+‐*^ *BINDING PROTEIN 1, GLUTAMATE RECEPTOR 2.5*), a number of genes encoding transcription factors (*DEHYDRATION RESPONSE ELEMENT B1A (DREBB1A)*, *WRKY DNA‐BINDING PROTEIN 55, HEAT SHOCK TRANSCRIPTION FACTOR A2, CALCIUM‐BINDING TRANSCRIPTION FACTOR NIG1*), as well as stress and hormone‐related genes that act downstream in the signaling cascade (*GALACTINOL SYNTHASE 2, LATE EMBRYOGENESIS ABUNDANT PROTEIN, TERPENOID CYCLASES FAMILY PROTEIN LUP5, INDOLE‐3‐ACETATE BETA‐D‐GLUCOSYLTRANSFERASE, and HEAT SHOCK PROTEIN 17.6A*) (Omidbakhshfard et al., 2020; Rasul et al., 2021). Altogether, this not only identifies several transcription factors presumably involved in the global transcriptional reprogramming by biostimulants but also implicates Ca^2+^ signaling, hormone, and heat shock pathways as modulators of the intricate biostimulant stress‐signaling network.

In tomato, molecular priming by *A. nodosum*‐derived biostimulant resulted in growth promotion, increased yield, and improved marketable grade of tomato fruits (Kanojia et al., 2024). This was associated with upregulation of genes encoding transcription factors from similar families (WRKY, NAC) both in leaves and the fruits. The transcriptional reprogramming involved induction of many stress‐protective genes, whereas senescence‐related genes were repressed. Genes encoding components from the jasmonic acid and brassinosteroids pathways were among the most induced under severe stress (Kanojia et al., 2024), the latter similar to Arabidopsis. This indicates that similar pathways (e.g., ABA, brassinosteroids) may be activated in both model and crop plants by the molecular priming.

Another brown seaweed biostimulant, conferring cold stress tolerance in tomato as well as larger and more fruits under both normal and cold stress conditions, induced genes related to proline, flavonoids, and chlorophyll (Borella et al., 2023). This transcriptomic data were corroborated by higher proline, polyphenols, flavonoids, and carotenoids contents in the biostimulant‐treated plants (Borella et al., 2023).

Other types of biostimulants triggered transcriptional reprogramming as well. In cotton, γ‐polyglutamic acid mediated drought tolerance through transcriptional reprogramming which included upregulation of several transcription factors from 25 different families, among which ERF, WRKY, NAC, and MYB were the most abundant (Wang et al., 2025). For example, *GhA03G01081* and *GhA05G00761*, highly induced by the biostimulant, were homologs of the Arabidopsis drought responsive ERF transcription factors from the DERB family. Consistent with that and similar to Arabidopsis, genes involved in several hormone pathways, for example, brassinosteroid, ethylene, and ABA pathways, were also transcriptionally regulated. Genes encoding the ABA receptor PYR (PYRACTIN)/PYL (PYR1‐LIKE), the downstream serine/threonine protein kinase SnRK2, and MAP3K17/18 were upregulated by the biostimulant, whereas a gene encoding a protein phosphatase PP2C, a negative regulator of ABA signaling, was repressed (Wang et al., 2025).

In maize, the molecular priming by chitosan‐fulvic acid nanoparticles, which enhanced tolerance to drought, induced genes encoding similar transcription factors, such as *ZmDREB1A*, *ZmNAC28, ZmbZIP1*, and the ABA‐dependent *ZmCIPK3* (Brown et al., 2024). This further corroborated the role of the ABA pathway in attaining drought tolerance by biostimulant priming.

A biological stimulant derived from a mixture of mycorrhizal microorganisms and rhizobacteria had a positive effect on tomato seedling plant growth and development under standard conditions and in response to moderate salinity (Justamante et al., 2025). The transcriptomic analysis showed differential expression of stress‐related genes such as *GOX3* or *DIR1*, as well as genes related to jasmonic acid and auxin hormones. A number of genes encoding transcription factors (TFs) from *WRKY* and other families, including TFs presumably involved in stress defense, were induced by the biostimulant with and without stress, suggesting that the biostimulant‐treated plants have already activated their defense system before the onset of stress (Justamante et al., 2025).

## STRATEGIES TO IDENTIFY NEW PLAYERS OF THE GENETIC NETWORK WHICH CONTROLS BIOSTIMULANT‐INDUCED STRESS TOLERANCE AND CROP YIELD ENHANCEMENT

As indicated above, biostimulants influence growth and development, as well as metabolism, stress responses, fruit size and quality, and ultimately yield (Dias et al., 2024; Ferreira et al., 2025; Hasanuzzaman, Raihan, Nowroz, & Nahar, 2023; Hasanuzzaman, Raihan, Siddika, et al., 2023; Kanojia et al., 2024; Omidbakhshfard et al., 2020; Pereira et al., 2025; Rasul et al., 2021; Staykov et al., 2020). These processes themselves are regulated by intricate genetic networks converging developmental and environmental signals, and utilize plant hormones, reactive oxygen species, lipids, and other secondary messengers (Gechev et al., 2006; Zhu, 2002). Whereas some of the stress signaling pathways are modulated by biostimulants, others are not related to biostimulants and act in alternative pathways. Despite the recent omics studies implicating particular genes and biochemical pathways in the molecular priming, no key genes have been identified and evaluated functionally so far. Below we describe strategies to functionally validate genes and pathways involved specifically in the biostimulant‐controlled genetic networks in both model plants (*A. thaliana*) and vegetable crops (pepper and tomato).

### Identification of genes that are part of the biostimulants signaling network in *Arabidopsis thaliana*

**Figure 1:**
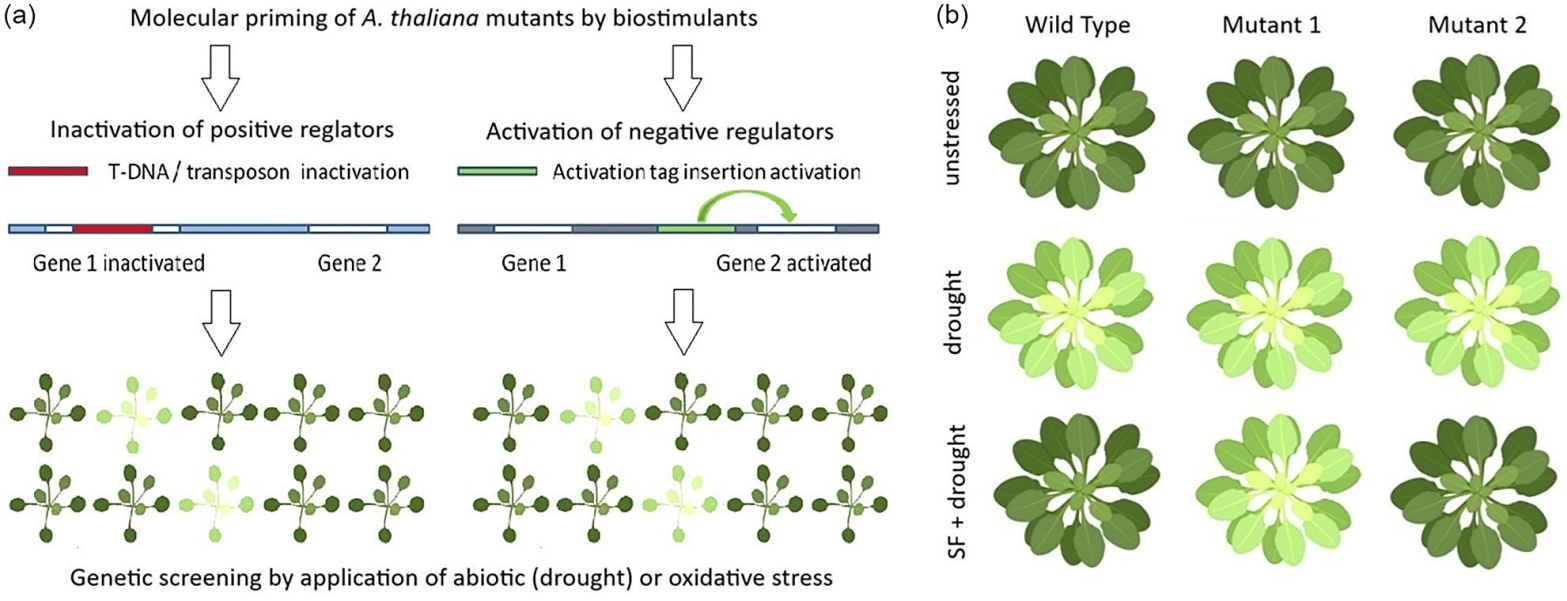
Mutant screening to identify genes, which are part of the biostimulant network. (a) Forward genetics approach utilizing either gene inactivation by T‐DNA/transposons to inactivate unknown positive regulators (gene 1) or activation tag construct to identify negative regulators (gene 2) of the biostimulant network. (b) Reverse genetic approach, in which genes regulated by biostimulants (e.g., SuperFifty, SF) are mutated or CRISPR‐Cas9 genome edited. If a gene is involved in the biostimulant pathway, its inactivation will result in a sensitive phenotype upon SF‐drought exposure (mutant 1), as opposed to the inactivation of a gene which is not part of the biostimulant pathway (mutant 2).

Biostimulants can protect Arabidopsis from many stresses such as drought, heat, and oxidative stress (Omidbakhshfard et al., 2020; Rasul et al., 2021; Staykov et al., 2020). However, when essential genes of the stress‐signaling network modulated by biostimulants are mutated, the biostimulants may no longer provide protection against abiotic and oxidative stresses, and their stress‐protective effect will be minimized. In contrast, mutations in genes that are not part of pathways modulated by biostimulants will not have an effect on the stress mitigation by the biostimulants. Hence, genetic studies can be designed to identify genes specifically involved in the biostimulants‐modulated gene network, using, for example, the well‐established system based on the protective effect of seaweed biostimulants against drought and oxidative stress (Omidbakhshfard et al., 2020; Rasul et al., 2021; Figure 1). Two alternative strategies can be applied to identify such biostimulant‐related genes:

#### Forward genetics screening of mutants to identify genes which are modulators of the biostimulant network

Large mutant collections of *A. thaliana* with genome coverage, containing mutants in nearly all Arabidopsis genes, can be screened to identify mutant lines which are sensitive to drought or/or oxidative stress after biostimulant application (Figure 1).

This is a labor‐costly but unbiased forward genetics approach, which will identify many new genes of the biostimulants stress‐protective network. After treatment with the biostimulant, mutated genes which are positive regulators of the biostimulant pathways will render plants sensitive to drought and oxidative stress (Figure 1). The mutant collections to be utilized can be both from chemical (EMS) or insertion (T‐DNA, transposons) mutagenesis. Negative regulators of the biostimulant pathways, on the other hand, can be identified by activation tag mutant collections, in which the activation tag constructs inserted near the negative regulators will activate them and consequently the activated repressors will suppress the biostimulant pathway (Figure 1).

#### Reverse genetics approach as a tool to validate genes from the biostimulant network

In this approach, comprehensive transcriptional analysis of biostimulants‐modulated gene expression, conducted by RNA sequencing, can identify genes that are regulated during biostimulant‐induced molecular priming. Selected biostimulant‐induced genes, presumably involved in the biostimulant network and contributing to the observed stress tolerance, will be inactivated by insertion mutagenesis (e.g., T‐DNA knockouts or transposons) or CRISPR‐Cas9 genome editing, and the resulting mutant and genome‐edited lines will be functionally evaluated for altered responses to biostimulants. The corresponding mutated/genome‐edited lines will be subjected to drought and oxidative stress to evaluate the functionality of the studied genes (Figure 1). Inactivation of genes that are positive modulators of the biostimulant pathways will presumably abolish the stress‐protective effect of the biostimulant on stress tolerance.

This reverse genetics approach offers a more targeted way to identify directly new genes that are essential players of the drought or oxidative stress‐signaling network. As oxidative stress is a consequence of many other abiotic stresses such as extreme temperatures and salinity, the mutant and genome‐edited lines will be evaluated for the whole spectrum of most prominent abiotic stresses (drought, heat, salt stress etc.). Altogether, the two approaches proposed by us have the potential to reveal the majority of the key biostimulant network genes in Arabidopsis.

### GWAS analysis of biostimulants response in major crops

The reverse genetics approach can be applied not only for Arabidopsis but also for major crops. Genes commonly regulated by the biostimulant in cereal, berry, and vegetable crops can be identified, and a selected number of them functionally validated (e.g., by CRISPR‐Cas9 genome editing in maize and tomato or VIGS in pepper). In this line, comprehensive transcriptome analyses by RNA‐seq of SuperFifty‐induced stress protection have very recently been conducted in maize, raspberry, and tomato (Kanojia et al., 2024; Kazakov et al., 2024). This will allow identification of key genes that contribute to agronomically important traits in these crops, including stress tolerance, corn and seed cob weight, fleshy fruit size and weight, and yield.

**Figure 2:**
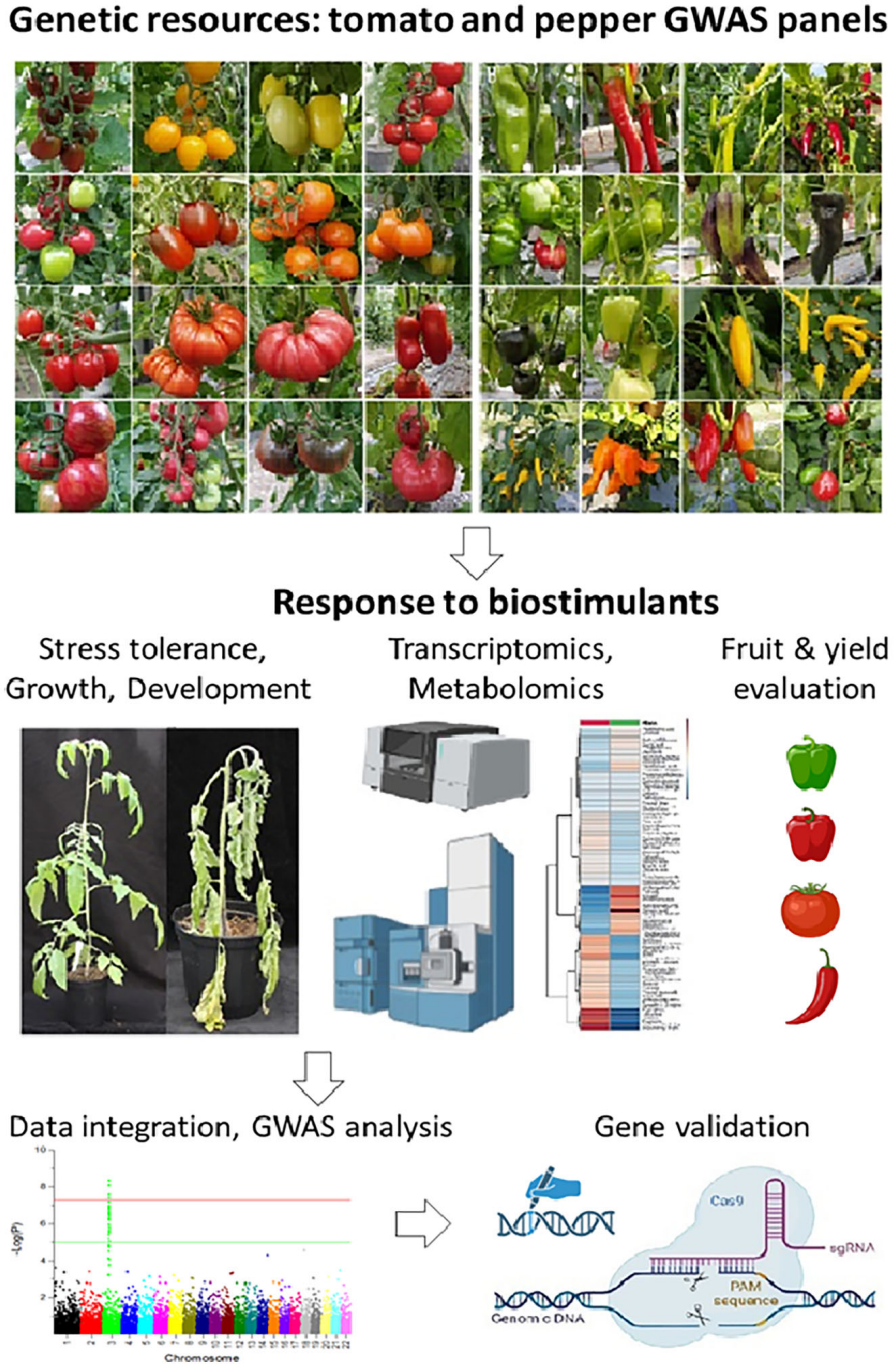
GWAS analysis pipeline for identification and verification of genes modulating the biostimulant pathway in vegetable crops. Genetic resources: hundreds of pepper and tomato lines and cultivars will be primed with biostimulants and evaluated under normal and stress (e.g., drought) conditions. Agronomically important traits such as growth, stress tolerance, fruit nutritional properties (metabolites, essential elements, vitamins, and yield) will be studied, and candidate genes identified by GWAS analysis will be verified using CRISPR‐Cas9 genome editing.

In an alternative approach, there is an exciting prospect to apply GWAS on the response of large panels of crop lines to biostimulant treatment, in order to identify genetic polymorphisms and corresponding genes that regulate processes such as biostimulant‐induced drought stress tolerance (Figure 2). The natural genetic variation has already been linked to different levels of stress tolerance and other agronomically important traits in tomato and other crops (Li et al., 2015; Ma et al., 2015; Mao et al., 2019; Wang et al., 2016; Zhang et al., 2017).

The GWAS approach proposed by us will identify naturally occurring genetic variations, which alter the responsiveness of pepper and tomato to biostimulants and influence their stress tolerance. The identified candidate genes will be verified by functional studies using CRISPR‐Cas9 genome editing or virus‐induced gene silencing, and the molecular mechanisms of biostimulant‐induced stress mitigation will be further studied by omics technologies such as transcriptomic and metabolomics (Figure 2). This GWAS approach has never been used with biostimulants and crops before. It will provide the researchers with tools to discover genes involved not only in stress tolerance but also genes, which modulate crop productivity and yield under unfavorable environmental conditions. As already mentioned before, drought and heat are the two most predominant abiotic stress factors influencing the world's agriculture, and the identification of genes that mitigate these stresses will have a profound impact on food security.

## PROSPECTS FOR ACHIEVING STRESS TOLERANCE AND YIELD ENHANCEMENT

Besides the fundamental value of the biostimulants research, which will reveal essential players in processes such as stress physiology, vegetative, and fruit development, the studies on the genetic control of biostimulant pathways will have a deep impact on global agriculture. As already noted, various stresses can account for more than 80% of agricultural production worldwide. The advantages of biostimulants allow broad application of this molecular priming technology to all major crops. Data that is already available from all major berry, cereal, and vegetable crops indicate that the biostimulants‐induced molecular priming has the potential to increase the revenues of farmers by either protection from unfavorable environmental conditions or/and enhancement of yield and crop quality (Dias et al., 2024; Ferreira et al., 2025; Hasanuzzaman, Raihan, Nowroz, & Nahar, 2023; Hasanuzzaman, Raihan, Siddika, et al., 2023; Jacomassi et al., 2022; Kanojia et al., 2024; Kazakov et al., 2024; Omidbakhshfard et al., 2020; Pereira et al., 2025; Rasul et al., 2021; Staykov et al., 2020; Sujata et al., 2023).

Clear examples of this positive impact on agriculture include the improved nitrogen uptake by an *A. nodosum*‐based biostimulant in Arabidopsis, wheat, and barley, with surplus N assimilated by the biomass in the form of glutamate, glutamine, free amino acids, soluble proteins, and chlorophyll (Goñi et al., 2021; Łangowski et al., 2022). Other examples include mitigation of heavy metal stress and salt stress in rice (Hasanuzzaman, Raihan, Nowroz, & Nahar, 2023; Hasanuzzaman, Raihan, Siddika, et al., 2023; Shahzad et al., 2023), as well as mitigation of salt stress and improvement of marketable grade and yield in tomato and other vegetable crops (Di Stasio et al., 2020; Kanojia et al., 2024). Di Stasio et al. (2020) reported that the seaweed biostimulant SuperFifty increased the number of tomato fruits and improved tomato yield by 70% under normal conditions. Furthermore, tomato plants primed with SuperFifty had larger fruits than unprimed plants under salt stress (Di Stasio et al., 2020). Likewise, Kanojia et al. (2024) reported larger fruits and higher yield in SuperFifty‐primed tomato, as well as mitigation of drought stress (Kanojia et al., 2024). Improving both stress tolerance and yield in tomato and other vegetable crops will have a huge impact on world agriculture and further contribute to global food security.

Furthermore, the GWAS studies on genetic resources in vegetable crops and their responses to biostimulants can reveal additional genes that modulate fruit size, metabolic content, and yield.

In berry crops, such as raspberry and strawberry, the molecular priming technology by seaweed biostimulants has the potential to increase the yield considerably, as indicated earlier (Table 1). Considering the volume of global raspberry and strawberry markets, 1.28 Bln EUR for raspberry and 17 Bln EUR for strawberry can be obtained as additional revenue from the enhanced production of berry fruits (Kazakov et al., 2024).

The perspectives of such studies are not limited to berry, cereal, and vegetable crops. The growth or/and stress tolerance of other crops, such as *Brassica juncea*, grapevine, okra (*Abelmoschus esculentus* L.), olive, papaya, and sugarcane, was also positively influenced by biostimulants (Dias et al., 2024; Ferreira et al., 2025; Jacomassi et al., 2022; Sujata et al., 2023). The molecular priming technology developed on the basis of seaweed biostimulants is widely applicable to all crops. Overall, it can be developed into a universal eco‐friendly technology that can protect all plants against the major abiotic stresses, as well as improve marketable grade and enhance crop yield.

## CONCLUSION AND OUTSTANDING QUESTIONS

There is growing evidence that biostimulants regulate many aspects of plant growth and development, as well as stress responses. Furthermore, many publications in recent years document the positive effect of the biostimulants on nutritional properties and crop yield. Comprehensive transcriptional profiling in model (*A. thaliana*) and crop plants implicates genes encoding signaling compounds, plant hormones such as ABA, brassinosteroids, and ethylene, and a number of transcription factors responsible for the global transcriptional reprogramming and the positive effects of the biostimulants. However, there are still no functional studies linking specific genes to the above‐mentioned processes. There is a huge perspective for research in this field to unravel the key genes of the apparently intricate biostimulants network. Some of the outstanding questions are: How do biostimulants regulate plant growth and development? How does the intricate plant stress network integrate with the biostimulants network to provide protection against a multitude of abiotic stresses? Is the biostimulants‐induced molecular priming universal in all crops? Which are the key biostimulant‐regulated genes that determine fruit quality and crop yield? Are these complex metabolic regulators or/and specific transcription factors? This will have important agronomical applications, for example, enhancing crop durability and productivity.

## CONFLICT OF INTEREST

The author declares no conflict of interest.

## Data Availability

The data that support the findings of this study are available on request from the corresponding author. The data are not publicly available due to privacy or ethical restrictions.

## References

[tpj70382-bib-0001] Alam, M.Z. , Braun, G. , Norrie, J. & Hodges, D.M. (2013) Effect of Ascophyllum extract application on plant growth, fruit yield and soil microbial communities of strawberry. Canadian Journal of Plant Science, 93, 23–36.

[tpj70382-bib-0002] Alghabari, F. & Shah, Z.H. (2025) Deciphering salt tolerance mechanisms in synthetic hexaploid and bread wheat under humic acid application: physiological and genetic perspectives. Frontiers in Plant Science, 16, 1545835. 40115959 10.3389/fpls.2025.1545835 PMC11922714

[tpj70382-bib-0003] Ali, J. , Jan, I. , Ullah, H. , Ahmed, N. , Alam, M. , Ullah, R. et al. (2022) Influence of *Ascophyllum nodosum* extract foliar spray on the physiological and biochemical attributes of okra under drought stress. Plants, 11(6), 790. 35336672 10.3390/plants11060790 PMC8949179

[tpj70382-bib-0004] Ali, S. , Akhtar, M.S. , Siraj, M. & Zaman, W. (2024) Molecular communication of microbial plant biostimulants in the rhizosphere under abiotic stress conditions. International Journal of Molecular Sciences, 25(22), 12424. 39596488 10.3390/ijms252212424 PMC11595105

[tpj70382-bib-0005] Bellotti, G. , Cortimiglia, C. , Antinori, M.E. , Cocconcelli, P.S. & Puglisi, E. (2025) Comprehensive genome‐wide analysis for the safety assessment of microbial biostimulants in agricultural applications. Microbial Genomics, 11(4), 001391. 40294085 10.1099/mgen.0.001391 PMC12038027

[tpj70382-bib-0006] Borella, M. , Baghdadi, A. , Bertoldo, G. , Della Lucia, M.C. , Chiodi, C. , Celletti, S. et al. (2023) Transcriptomic and physiological approaches to decipher cold stress mitigation exerted by brown‐seaweed extract application in tomato. Frontiers in Plant Science, 14, 1232421. 37767293 10.3389/fpls.2023.1232421 PMC10520554

[tpj70382-bib-0007] Brown, A. , Al‐Azawi, T.N.I. , Methela, N.J. , Rolly, N.K. , Khan, M. , Faluku, M. et al. (2024) Chitosan‐fulvic acid nanoparticles enhance drought tolerance in maize via antioxidant defense and transcriptional reprogramming. Physiologia Plantarum, 176(4), e14455. 39073158 10.1111/ppl.14455

[tpj70382-bib-0008] Di Gaudio, F. , Vasto, S. , Sabatino, L. , Ferrantelli, V. , Macaluso, A. , Caldarella, R. et al. (2025) Consumption of lettuce with seaweed extract biostimulant application improved iron homeostasis in a randomized interventional trial of healthy individuals. Scientific Reports, 15(1), 7799. 40050333 10.1038/s41598-025-91380-7 PMC11885599

[tpj70382-bib-0009] Di Sario, L. , Boeri, P. , Matus, J.T. & Pizzio, G.A. (2025) Plant Biostimulants to Enhance Abiotic Stress Resilience in Crops. International Journal of Molecular Sciences, 26(3), 1129. 39940896 10.3390/ijms26031129 PMC11817731

[tpj70382-bib-0010] Di Stasio, E. , Cirillo, V. , Raimondi, G. , Giordano, M. , Esposito, M. & Maggio, A. (2020) Osmo‐priming with seaweed extracts enhances yield of salt‐stressed tomato plants. Agronomy, 10, 1559.

[tpj70382-bib-0011] Dias, M.C. , Figueiras, R. , Sousa, M. , Araújo, M. , de Oliveira, J.M.P.F. , Pinto, D.C.G.A. et al. (2024) *Ascophyllum nodosum* extract improves olive performance under water deficit through the modulation of molecular and physiological processes. Plants, 13(20), 2908. 39458857 10.3390/plants13202908 PMC11511455

[tpj70382-bib-0012] Dutta, S.K. , Layek, J. , Yadav, A. , Das, S.K. , Rymbai, H. , Mandal, S. et al. (2023) Improvement of rooting and growth in kiwifruit (*Actinidia deliciosa*) cuttings with organic biostimulants. Heliyon, 9(7), e17815. 37455949 10.1016/j.heliyon.2023.e17815 PMC10339021

[tpj70382-bib-0013] El‐Hefny, M. & Hussien, M.K. (2025) Enhancing the growth and essential oil components of *Lavandula latifolia* using *Malva parviflora* extract and humic acid as biostimulants in a field experiment. Scientific Reports, 15(1), 774. 39755703 10.1038/s41598-024-82127-x PMC11700101

[tpj70382-bib-0014] Ferreira, T.R. , Rodrigues, J.D.S. , Galote, J.K.B. , Crasque, J. , Neto, B.C. , Falqueto, A.R. et al. (2025) Mitigation of high temperatures with *Ascophyllum nodosum* biostimulants in papaya (*Carica papaya L*.) seedlings. Plants, 14(3), 317. 39942879 10.3390/plants14030317 PMC11821136

[tpj70382-bib-0015] Gatti, N. , Maghrebi, M. , Serio, G. , Gentile, C. , Bunea, V.V. , Vigliante, I. et al. (2025) Seaweed and yeast extracts as sustainable phytostimulant to boost secondary metabolism of apricot fruits. Frontiers in Plant Science, 15, 1455156. 39925374 10.3389/fpls.2024.1455156 PMC11802282

[tpj70382-bib-0016] Gechev, T.S. , Van Breusegem, F. , Stone, J.M. , Denev, I. & Laloi, C. (2006) Reactive oxygen species as signals that modulate plant stress responses and programmed cell death. BioEssays, 28(11), 1091–1101. 17041898 10.1002/bies.20493

[tpj70382-bib-0017] Goñi, O. , Łangowski, Ł. , Feeney, E. , Quille, P. & O'Connell, S. (2021) Reducing nitrogen input in barley crops while maintaining yields using an engineered biostimulant derived from *Ascophyllum nodosum* to enhance nitrogen use efficiency. Frontiers in Plant Science, 12, 664682. 34025702 10.3389/fpls.2021.664682 PMC8132967

[tpj70382-bib-0018] Guinan, K.J. , Sujeeth, N. , Copeland, R.B. , Jones, P.W. , O'Brien, N.M. , Sharma, H.S.S. et al. (2013) Discrete roles for extracts of *Ascophyllum nodosum* in enhancing plant growth and tolerance to abiotic and biotic stresses. Acta Horticulturae, 1009, 127–135.

[tpj70382-bib-0019] Harashima, H. , Dissmeyer, N. & Schnittger, A. (2013) Cell cycle control across the eukaryotic kingdom. Trends in Cell Biology, 23, 345–356. 23566594 10.1016/j.tcb.2013.03.002

[tpj70382-bib-0020] Hasanuzzaman, M. , Raihan, M.R.H. , Nowroz, F. & Nahar, K. (2023) Insight into the physiological and biochemical mechanisms of biostimulating effect of *Ascophyllum nodosum* and *Moringa oleifera* extracts to minimize cadmium‐induced oxidative stress in rice. Environmental Science and Pollution Research International, 30(19), 55298–55313. 36890405 10.1007/s11356-023-26251-7

[tpj70382-bib-0021] Hasanuzzaman, M. , Raihan, M.R.H. , Siddika, A. , Rahman, K. & Nahar, K. (2023) Supplementation with *Ascophyllum nodosum* extracts mitigates arsenic toxicity by modulating reactive oxygen species metabolism and reducing oxidative stress in rice. Ecotoxicology and Environmental Safety, 255, 114819. 36963188 10.1016/j.ecoenv.2023.114819

[tpj70382-bib-0022] Huy, V.N. , Methela, N.J. , Al‐Azawi, T.N.I. , Khan, M. , Faluku, M. , Brown, A. et al. (2025) Fulvic acid‐releasing chitosan nanoparticles promote the growth and salt stress tolerance of soybean plants. Physiologia Plantarum, 177(3), e70254. 40325609 10.1111/ppl.70254 PMC12053295

[tpj70382-bib-0023] Jacomassi, L.M. , Viveiros, J.O. , Oliveira, M.P. , Momesso, L. , de Siqueira, G.F. & Crusciol, C.A.C. (2022) A seaweed extract‐based biostimulant mitigates drought stress in sugarcane. Frontiers in Plant Science, 13, 865291. 35574093 10.3389/fpls.2022.865291 PMC9096543

[tpj70382-bib-0024] Jasso‐Robles, F.I. , Aucique‐Perez, C.E. , Zeljković, S.Ć. , Saiz‐Fernández, I. , Klimeš, P. & De Diego, N. (2024) The loss‐of‐function of AtNATA2 enhances AtADC2‐dependent putrescine biosynthesis and priming, improving growth and salinity tolerance in Arabidopsis. Physiologia Plantarum, 176(6), e14603. 39489618 10.1111/ppl.14603 PMC11659803

[tpj70382-bib-0025] Justamante, M.S. , Larriba, E. , Zavala‐González, E.A. , Aranda‐Martínez, A. & Pérez‐Pérez, J.M. (2025) Transcriptional profiling to assess the effects of biological stimulant Atlanticell Micomix on tomato seedlings under salt stress. Plants, 14(8), 1198. 40284086 10.3390/plants14081198 PMC12030531

[tpj70382-bib-0026] Kanojia, A. , Lyall, R. , Sujeeth, N. , Alseekh, S. , Martínez‐Rivas, F. , Fernie, A.R. et al. (2024) Physiological and molecular insights into the effect of a seaweed biostimulant on enhancing fruit yield and drought tolerance in tomato. Plant Stress, 14, 100692.

[tpj70382-bib-0027] Kazakov, P. , Alseekh, S. , Ivanova, V. & Gechev, T. (2024) Biostimulant‐based molecular priming improves crop quality and enhances yield of raspberry and strawberry fruits. Metabolites, 14(11), 594. 39590830 10.3390/metabo14110594 PMC11596280

[tpj70382-bib-0028] Kerchev, P. , van der Meer, T. , Sujeeth, N. , Verlee, A. , Stevens, C.V. , Van Breusegem, F. et al. (2020) Molecular priming as an approach to induce tolerance against abiotic and oxidative stresses in crop plants. Biotechnology Advances, 40, 107503. 31901371 10.1016/j.biotechadv.2019.107503

[tpj70382-bib-0029] Łangowski, Ł. , Goñi, O. , Ikuyinminu, E. , Feeney, E. & O'Connell, S. (2022) Investigation of the direct effect of a precision *Ascophyllum nodosum* biostimulant on nitrogen use efficiency in wheat seedlings. Plant Physiology and Biochemistry, 179, 44–57. 35306329 10.1016/j.plaphy.2022.03.006

[tpj70382-bib-0030] Lengrand, S. , Dubois, B. , Pesenti, L. , Debode, F. & Legrève, A. (2024) Humic substances increase tomato tolerance to osmotic stress while modulating vertically transmitted endophytic bacterial communities. Frontiers in Plant Science, 15, 1488671. 39628527 10.3389/fpls.2024.1488671 PMC11611569

[tpj70382-bib-0031] Li, X.M. , Chao, D.Y. , Wu, Y. , Huang, X.H. , Chen, K. , Cui, L.G. et al. (2015) Natural alleles of a proteasome alpha 2 subunit gene contribute to thermotolerance and adaptation of African rice. Nature Genetics, 47, 827. 25985140 10.1038/ng.3305

[tpj70382-bib-0032] Ma, Y. , Dai, X. , Xu, Y. , Luo, W. , Zheng, X. , Zeng, D. et al. (2015) COLD1 confers chilling tolerance in rice. Cell, 160, 1209–1221. 25728666 10.1016/j.cell.2015.01.046

[tpj70382-bib-0033] Makonya, G.M. , Bryla, D.R. , Hardigan, M.A. , Hoashi‐Erhardt, W. & DeVetter, L.W. (2025) Biostimulants with glycine betaine or kelp extract alleviate heat stress in red raspberry (*Rubus idaeus*). Scientific Reports, 15(1), 2251. 39824907 10.1038/s41598-024-83955-7 PMC11742384

[tpj70382-bib-0034] Mao, D.H. , Xin, Y.Y. , Tan, Y.J. , Hu, X.J. , Bai, J.J. , Liu, Z.Y. et al. (2019) Natural variation in the HAN1 gene confers chilling tolerance in rice and allowed adaptation to a temperate climate. PNAS USA, 116, 3494–3501. 30808744 10.1073/pnas.1819769116 PMC6397538

[tpj70382-bib-0035] Mishra, A. , Kar, S. , Bisht, N. , Mishra, S.K. & Chauhan, P.S. (2025) Synergistic effect of *Adathoda vasica* plant‐derived biostimulant and PGPR on *Zea mays* L. for drought stress management. Microbiological Research, 290, 127968. 39536514 10.1016/j.micres.2024.127968

[tpj70382-bib-0036] Mohamed, M.H.M. , Ali, M.M.E. , Zewail, R.M.Y. , Liava, V. & Petropoulos, S.A. (2024) Response of purslane plants grown under salinity stress and biostimulant formulations. Plants, 3(17), 2431. 10.3390/plants13172431 PMC11397487 39273915

[tpj70382-bib-0037] Monteiro, E. , Baltazar, M. , Pereira, S. , Correia, S. , Ferreira, H. , Alves, F. et al. (2023) *Ascophyllum nodosum* extract and glycine betaine preharvest application in grapevine: Enhancement of berry quality, phytochemical content and antioxidant properties. Antioxidants, 12(10), 1835. 37891914 10.3390/antiox12101835 PMC10603969

[tpj70382-bib-0038] Monteiro, E. , De Lorenzis, G. , Ricciardi, V. , Baltazar, M. , Pereira, S. , Correia, S. et al. (2024) Exploring seaweed and glycine betaine biostimulants for enhanced phenolic content, antioxidant properties, and gene expression of *Vitis vinifera* cv. “Touriga Franca” berries. International Journal of Molecular Sciences, 25(10), 5335. 38791373 10.3390/ijms25105335 PMC11121377

[tpj70382-bib-0039] Muradoğlu, F. , Batur, Ş. , Hasanov, M. & Güler, E. (2025) Putrescine eases saline stress by regulating biochemicals, antioxidative enzymes, and osmolyte balance in hydroponic strawberries (cv. Albion). Physiologia Plantarum, 177(3), e70259. 40344251 10.1111/ppl.70259 PMC12062852

[tpj70382-bib-0040] Omidbakhshfard, M.A. , Sujeeth, N. , Gupta, S. , Omranian, N. , Guinan, K.J. , Brotman, Y. et al. (2020) A Biostimulant obtained from the seaweed *Ascophyllum nodosum* protects *Arabidopsis thaliana* from severe oxidative stress. International Journal of Molecular Sciences, 21, 474. 31940839 10.3390/ijms21020474 PMC7013732

[tpj70382-bib-0041] Osman, H.S. , Gao, Y. , Luo, Z. , Alharbi, K. , Rashwan, E. , Omara, A.E. et al. (2025) Integrative use of biochar and biostimulants improves cadmium detoxification and yield in cotton. Science of the Total Environment, 981, 179585. 40328069 10.1016/j.scitotenv.2025.179585

[tpj70382-bib-0042] Pereira, S. , Rodrigues, J. , Sujeeth, N. , Guinan, K.J. & Gonçalves, B. (2024) Optimizing strawberry growth: Impact of irrigation and biostimulant application on physiology and fruit quality. Plant Stress, 15, 100715.

[tpj70382-bib-0043] Pereira, S. , Silva, V. , Guedes, F. , Raimundo, F. , Sousa, J.R. , Silva, A.P. et al. (2024) Physiological and biochemical responses of ‘Burlat’ sweet cherry to pre‐harvest foliar application of calcium and seaweed extracts. Horticulturae, 10, 1173.

[tpj70382-bib-0044] Pereira, S.I.A. , Aroca, R. & Cornejo, P. (2025) Use of biostimulants in beneficial plant‐microbe interactions. Frontiers in Plant Science, 16, 1592681. 40235916 10.3389/fpls.2025.1592681 PMC11997714

[tpj70382-bib-0045] Pring, S. , Kato, H. , Taniuchi, K. , Camagna, M. , Saito, M. , Tanaka, A. et al. (2025) Mixed DAMP/MAMP oligosaccharides promote both growth and defense against fungal pathogens of cucumber. Plant Science, 359, 112578.40414359 10.1016/j.plantsci.2025.112578

[tpj70382-bib-0046] Rasul, F. , Gupta, S. , Olas, J.J. , Gechev, T. , Sujeeth, N. & Mueller‐Roeber, B. (2021) Priming with a seaweed extract strongly improves drought tolerance in Arabidopsis. International Journal of Molecular Sciences, 22, 1469. 33540571 10.3390/ijms22031469 PMC7867171

[tpj70382-bib-0047] Shahzad, R. , Harlina, P.W. , Gallego, P.P. , Flexas, J. , Ewas, M. , Leiwen, X. et al. (2023) The seaweed *Ascophyllum nodosum*‐based biostimulant enhances salt stress tolerance in rice (Oryza sativa L.) by remodeling physiological, biochemical, and metabolic responses. Journal of Plant Interactions, 18, 2266514.

[tpj70382-bib-0048] Song, J. , Pi, B. , Dai, J. , Nie, Z. , Yu, G. & Du, W. (2025) Effects of humic acid on the growth and cadmium accumulation of maize (*Zea mays* L.) seedlings. International Journal of Phytoremediation, 27(7), 888–895. 39838591 10.1080/15226514.2025.2455483

[tpj70382-bib-0049] Staykov, N.S. , Angelov, M. , Petrov, V. , Minkov, P. , Kanojia, A. , Guinan, K.J. et al. (2020) An *Ascophyllum nodosum*‐derived biostimulant protects model and crop plants from oxidative stress. Metabolites, 11, 24. 33396419 10.3390/metabo11010024 PMC7824492

[tpj70382-bib-0050] Sujata, V.G. , Baliyan, V. , Avtar, R. & Mehrotra, S. (2023) Alleviating drought stress in *Brassica juncea* (L.) Czern & Coss. by foliar application of biostimulants‐orthosilicic acid and seaweed extract. Applied Biochemistry and Biotechnology, 195(1), 693–721. 35986841 10.1007/s12010-022-04085-2

[tpj70382-bib-0051] Sujeeth, N. , Petrov, V. , Guinan, K.J. , Rasul, F. , O'Sullivan, J.T. & Gechev, T.S. (2022) Current insights into the molecular mode of action of seaweed‐based biostimulants and the sustainability of seaweeds as raw material resources. International Journal of Molecular Sciences, 23(14), 7654. 35886998 10.3390/ijms23147654 PMC9318209

[tpj70382-bib-0052] Takahashi, I. , Kojima, S. , Sakaguchi, N. , Umeda‐Hara, C. & Umeda, M. (2010) Two Arabidopsis cyclin A3s possess G1 cyclin‐like features. Plant Cell Reports, 29, 307–315. 20130883 10.1007/s00299-010-0817-9

[tpj70382-bib-0053] Velásquez, A.C. , Castroverde, C.D.M. & He, S.Y. (2018) Plant–pathogen warfare under changing climate conditions. Current Biology, 28, R619–R634. 29787730 10.1016/j.cub.2018.03.054 PMC5967643

[tpj70382-bib-0054] Wang, X.L. , Wang, H.W. , Liu, S.X. , Ferjani, A. , Li, J.S. , Yan, J.B. et al. (2016) Genetic variation in ZmVPP1 contributes to drought tolerance in maize seedlings. Nature Genetics, 48, 1233–1241. 27526320 10.1038/ng.3636

[tpj70382-bib-0055] Wang, Z. , Zhang, X. , Liusui, Y. , Fu, W. , Han, A. , Zhao, D. et al. (2025) Unveiling the molecular mechanisms of gamma‐polyglutamic acid‐mediated drought tolerance in cotton through transcriptomic and physiological analyses. BMC Plant Biology, 25(1), 392. 40148798 10.1186/s12870-025-06406-z PMC11948946

[tpj70382-bib-0056] Zhang, L. , Freschi, G. , Rouphael, Y. , De Pascale, S. & Lucini, L. (2023) The differential modulation of secondary metabolism induced by a protein hydrolysate and a seaweed extract in tomato plants under salinity. Frontiers in Plant Science, 13, 1072782. 36726679 10.3389/fpls.2022.1072782 PMC9884811

[tpj70382-bib-0057] Zhang, Z.Y. , Li, J.J. , Pan, Y.H. , Li, J.L. , Zhou, L. , Shi, H.L. et al. (2017) Natural variation in CTB4a enhances rice adaptation to cold habitats. Nature Communications, 8, 14788. 10.1038/ncomms14788 PMC5376651 28332574

[tpj70382-bib-0058] Zhu, J.K. (2002) Salt and drought stress signal transduction in plants. Annual Review of Plant Biology, 53, 247–273. 10.1146/annurev.arplant.53.091401.143329 PMC3128348 12221975

